# Design of Innovative Divalent Cj1621 and CjaA Multiepitope mRNA-Based Vaccine Against Foodborne *Campylobacter jejuni* Using In Silico Approaches

**DOI:** 10.1155/vmi/3487209

**Published:** 2025-06-28

**Authors:** Dhama Al-Sallami, Amjed Alsultan, Amir Hani Raziq, Behrooz Sadeghi Kalani, Simon R. Clarke

**Affiliations:** ^1^Department of Physiology, Pharmacology and Biochemistry, College of Veterinary Medicine, University of Al-Qadisiyah, Al-Diwaniyah, Iraq; ^2^Department of Internal and Preventive Medicine, College of Veterinary Medicine, University of Al-Qadisiyah, Al-Diwaniyah, Iraq; ^3^Department of Medical Laboratory Diagnosis, College of Health Science, University of Duhok, Duhok, Iraq; ^4^Department of Microbiology, Faculty of Medicine, Ilam University of Medical Sciences, Ilam, Iran; ^5^School of Biological Sciences, University of Reading, Whiteknights, Reading RG6 6EX, UK

**Keywords:** *Campylobacter jejuni*, divalent construct, immunoinformatic, multiepitope-based vaccine

## Abstract

*Campylobacter jejuni* is one of the main causes of gastroenteritis in human and animals worldwide. Emergence of antibiotic resistance in microorganisms increased the need to develop new types of vaccines. The present study aimed to design novel multiepitope mRNA vaccine against *Campylobacter jejuni* using immunoinformatics tools. For this purpose, two virulence *C. jejuni* proteins (Cj1621 and CjaA) were selected as antigen targets, and the appropriate epitopes were predicted using immunoinformatics tools and molecular models. Five cytotoxic T lymphocyte, six helper T lymphocyte, four linear B-cell, and one conformational B-cell epitopes were linked together with an appropriate linker, and then, adjuvant (RpfE) was attached to the construct candidate. Physiochemical, immunological, secondary, and 3D structure evaluation of the proposed vaccine showed it is immunogenic, nontoxic, nonallergic, flexible, and stable. Furthermore, docking shows that the vaccine has stable interaction with the immune receptors TLR (TLR-2 and TLR-4) and B7 (B7-1 and B7-2). Moreover, analysis of the vaccine with the MD server shows its ability to induce humoral and cellular immunity of the selected host. Overall, our findings indicate that the proposed vaccine could be a promising option against *Campylobacter jejuni* infection; however, further lab-based studies are needed to confirm the efficiency and safety of this vaccine.

## 1. Introduction

Bacterial infection is a real threat to the public health around the world in the recent years. The increasing bacterial resistance to different antibiotics has led to develop new types of vaccines [1]. *C. jejuni* is a Gram-negative, curved bacterium, microaerophilic, and considered the most common bacteria that cause foodborne enteritis in human and farm animals [2]. However, it is commensal in birds, especially chickens. *C. jejuni* causes high economic losses in human and domestic animals [3]. Interestingly, dose of infection with *C. jejuni* is various between human and birds. Only 500–800 bacteria can incident the infection in human [4]. Infection with *C. jejuni* is characterized by watery or bloody diarrhea with fever and abdominal pain [5, 6].

The efforts are continuous to develop safe and effective vaccine against *C. jejuni*. The normal attenuated and killed vaccine might induce autoantibodies causing Guillain–Barre Syndrome for some patients. This is due to the type of lipo-oligosaccharides on the surface of the *C*. *jejuni* cell wall [7]. Thus, subunit vaccine can be the alternative type of vaccine against *C. jejuni* infection [8]. Multiple epitope vaccine is a modern vaccine approach, and it allows good presentation to the antigen and no covalent binding between different branches of vaccine peptides [9]. In addition, it is safe and low cost [10]. Alkyl hydroperoxide reductase (AhpC), outer membrane protein (Omp), and Flagellar L-ring protein precursor (FlgH) are campylobacter proteins that were used as subunit vaccine against *Campylobacter* infection in previous studies [11–13].

The mRNA vaccines mark a significant advancement in the immunization technology by introducing a genetic material that prompts cells to produce specific proteins, triggering an immune response. Their rapid development and adaptability allow for quick responses to emerging pathogens, while the absence of live virus components enhances safety. The ability of mRNA vaccines induces humoral and cellular responses, making them effective against intracellular infections [14]. Additionally, improvement in bioinformatics and computational technologies has revolutionized drug and vaccine design. These tools enable researchers to analyze biological data, predict molecular interactions, and create novel compounds with high efficacy. This accelerates the development of treatments and vaccines, enhancing our ability to combat infectious diseases [15]. In the current study, preparation of multiepitope mRNA vaccine against *C. jejuni* has been performed, and construct of two important virulence genes (Cj1621 and CjaA) was generated. Cj1621 is cell membrane protein, specific in *Campylobacter* and expected to poses trans membrane domains and conformational epitope. It is a novel periplasmic protein and can be used as a vaccine candidate against campylobacter; it is important in *C. jejuni* diagnosis as well [16]. *C*. *jejuni* antigen A (CjaA) is a soluble binding protein and a part of the ABC transport system. It is extra cytoplasmic and necessary for in vivo colonization of *C. jejuni* [17]. Also, it is well known that amino acids are vital for campylobacter metabolism. In addition to the role of amino acids as the main source of nitrogen, they have varied attributions in physiology of this pathogen. CjaA protein could be behind carrying of cysteine and other amino acids as shown in the previous study. Further, CjaA is crucial in campylobacter colonization, especially under iron deficient conditions [18, 19].

## 2. Materials and Methods

**Figure 1:**
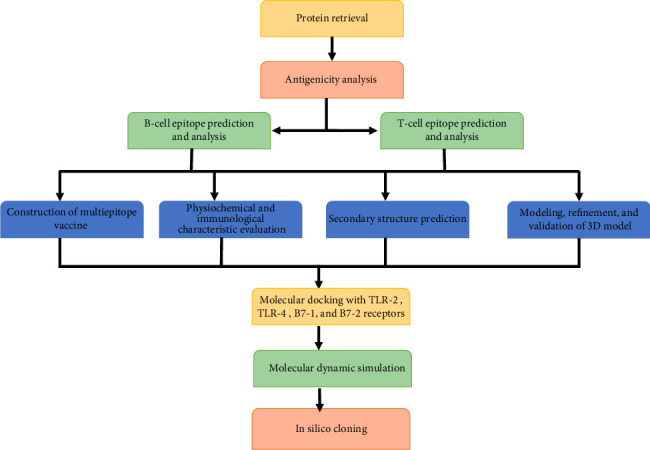
Chart represents the work flow of the study: The flowchart outlines the steps involved in the design of the proposed vaccine. In the first step, two virulence proteins were selected. Next, B-cell and T-cell epitopes were identified. Subsequently, these epitopes were assembled, and finally, the physicochemical and immuno properties of the proposed vaccine were evaluated.

The work flow of the present study is provided in Figure 1.

### 2.1. Sequence Retrieval of Protein

The UniProt Knowledgebase [20] was used for the retrieval of two selected proteins sequences of the *C. jejuni* NCTC 11168 strain (Cj1621 protein, accession number: Q0P807 and CjaA protein, accession number: A7H355).

### 2.2. Epitope Mapping

B-cell and T-cell epitopes were mapped by the IEDB server [21]. The high rank epitopes were candidate to construct of the vaccine candidate.

### 2.3. Human Homology

The NCBI BLASTp was used to check the homology between the predicated epitope with the homosapiens (TaxID: 9606) peptide database [22]. Epitopes with an E-value of 0.05 or higher are considered to have significant homology to the human peptide.

### 2.4. Evaluation of Allergenicity, Antigenicity, and Toxicity

Epitopes antigenicity was tested using VaxiJen v2.0 [23, 24]. Antigenicity was established on a 0.5 threshold. The highest antigenic epitopes were analyzed for allergenicity predictions using AllerTOP [25]. The toxicity prediction was performed by the ToxinPred web server [26].

### 2.5. Construction of the Multiepitope Vaccine Candidate

Epitopes were fused together to construct the proposed vaccine. Amino acid linker (GPGPG, AAY, and KK) were used to link the epitopes together. The resuscitation promoting factor E of *Mycobacterium tuberculosis* (UniProt ID: O53177) was selected as adjuvant and then fused to the construct via the EAAAK linker. In order to increase stability of the mRNA vaccine candidate, rabbit beta-globin and hemoglobin subunit beta (HBB) were added as UTR at C and N ends of the construct, respectively.

### 2.6. Physicochemical and Immunological Profile of the Vaccine Assemble

ProtParam [27] was used to estimate the physicochemical nature of the vaccine candidate. VaxiJen v2.0 and AllerTOP online tools were used to check the antigenicity and allergenicity of the vaccine construct, respectively.

### 2.7. Secondary Structure Prediction

The P-RoBi web server was used to predict the structural features of the proposed vaccine, such as alpha-helices, beta-turns, and regions of random coil [28].

### 2.8. 3D Structure Modeling Prediction, Refinement, and Validation

The 3D structure of the construct was predicted using the trRosetta server. [29]. The Galaxy Refine web server was used for refining of the proposed structure [30]. Ramachandran plot analysis was carried out using the RAMPAGE web server for the structure validation [31].

### 2.9. Molecular Docking

To estimate the affinity between the vaccine construct and receptor molecule, the ClusPro v2.0 server was used [32]. Also, ClusPro was applied to analyze the cooperation between the vaccine and receptor molecules. The TLR-2 (PDB ID 2Z80), TLR-4(PDB ID 2Z63), B7-1(PDB ID 1DR9), and B7-2(PDB ID 1NCN) receptors were picked and downloaded from the PDB web server.

### 2.10. Molecular Dynamic Simulation

Stability and motion of atoms within the complex (proposed vaccine and receptors) were predicted with the iMODS server [33], where the immune response can be elicited by the researcher via understanding dynamic behavior of the complex structures.

### 2.11. Vaccine 3D Assembly Flexibility

The CABS Flex 2.0 [34] which employs coarse-grained simulations to investigate protein dynamics was utilized to evaluate the structurally stable conformation of the proposed vaccine. Default parameters were applied for the distance restraint generator throughout the assessment.

### 2.12. Codon Optimization and mRNA Structure

Codon optimization is essential for efficient gene expression inside the foreign host. The JCat was used for codon optimization of the proposed vaccine [35]. Depending on guanine–cytosine (GC) content and the codon adaptation index, optimization of codon was generated. The secondary structure was predicted using the RNAfold server, providing insights into stability and function. In silico cloning was performed using the Snap Gene 4.2 tool [36].

## 3. Results

### 3.1. Sequence Retrieval and Antigenicity of the Vaccine Model

The selected proteins are important for colonization and amino acid metabolism in *C. jejuni*. Antigenicity of these proteins was estimated using the VaxiJen v2.0 server. Determination of the antigenicity was depended on a 0.5 threshold. The results show that the proposed vaccine is probable antigen with a 0.73 antigenic score.

### 3.2. Epitope Mapping

Only the antigenic, no allergic, nontoxic, and 100% conserved epitopes of Cj1621 and CjaA were selected, as shown in Tables 1, 2, and 3. In Table 1, five immunogenic, nontoxic, and nonallergenic cytotoxic T lymphocyte (CTL) epitopes were selected from Cj1621 and CjaA sequences to build up the candidate vaccine. Also, Table 2 shows that six helper T lymphocyte (HTL), four linear linear B-lymphocyte (LBL) epitopes (Table 3), and one B-conformational epitope were used to build the proposed vaccine (Table 4).

### 3.3. Construction of the Proposed Vaccine

**Figure 2:**
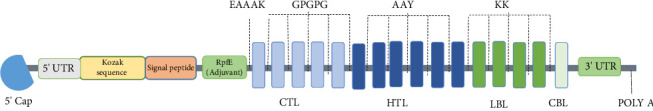
Graphical map of the mRNA multiepitope vaccine construct: HBB (UTR), Kozak sequence, signal peptide, and adjuvant were linked at the N terminal with help of the EAAAK linker. CTL fused via GPGPG, while HTL fused via AAY. The B cell fused via KK. Rabbit beta-globin was linked to the C terminal as UTR.

The vaccine candidate generates utilizing CTL, HTL, and LBL epitopes that were mentioned previously. As shown in Figure 2, the final construction of the mRNA vaccine candidate includes 5 m7GCap, HBB, Kozak sequence, SP, amino acid linker, RpfE, 15 B and T-cell epitopes, rabbit beta-globin, and poly A tail (Figure 2). AAY, GPGPG, and KK were used to link up the epitope's sequences together.

### 3.4. Physiochemical and Immunological Analysis of the Vaccine

VaxiJen, ANTIGENpro, and AllerTOP server were applied to estimate the immunogenicity of the candidate. Results show that the vaccine candidate is antigenic, nonallergic, and nontoxic to the host cell. The designed protein has a molecular weight of 26,198.64 Da; the chemical formula is C_1719_H_2657_N_477_O_515_S_2_; estimated half live in the mammalian cell is about 1.9 h, in bacteria is 10 h, and in yeast is 20 min in vivo. The theoretical isoelectric point is 9.51, and the grand average of hydropathicity is −0.587 (Table 5). Overall, the results reveal that the proposed vaccine is immunogenic and stable.

### 3.5. Secondary Structure of the Proposed Vaccine

**Figure 3:**
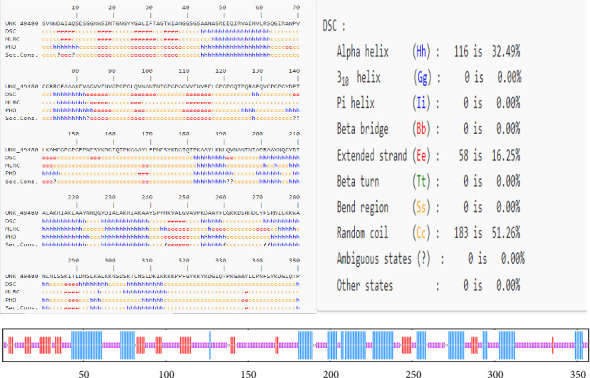
Prediction of secondary structure elements for the engineered mRNA multiepitope-based vaccine was performed using PSIPRED 4.0. The structural components are depicted as follows: pink for coils, blue for helices, and red for strands.

The secondary structure of the vaccine was predicted using the PRABI server. The constructed protein, comprising 357 amino acids, was found to contain 32.49% alpha helices, 16.25% extended strands, and 51.26% random coils (Figure 3). It was noticed that during the infection, the unfolded regions in the protein are detected by the antibodies.

### 3.6. Modeling, Refinement, and Validation of the 3D Model

**Figure 4:**
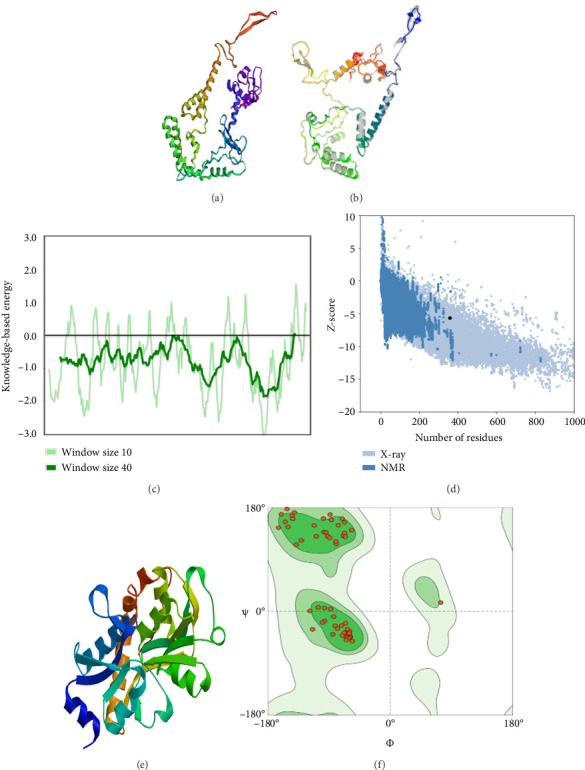
Predication and validation of the vaccine structure. (a) Three-dimensional model built of the vaccine candidate. (b) Refined the structure model by the galaxy web server. (c–f) Validation of the modeled structure. (c) The modeled structure that verified with the Z-score of −6.98. (d) Plot of the residue score. (e) 3D model of the vaccine. (f) Ramachandran favored and outliers were 97.26% and 0.35, respectively.

Out of the 5 models, Model 1 was selected as the tertiary structure of the proposed vaccine using trRosetta (Figure 4(b)). The Galaxy web tool was used to refine the selected structure (Figure 4(c)). The Z score that was estimated with ProSA was −5.68, while Ramachandran favored and outliers were 97.26% and 0.35, respectively, in SWISS-MODEL software simulation (Figures 4(d), 4(e), and 4(f)).

### 3.7. Molecular Docking and Molecular Dynamic Simulation

**Figure 5:**
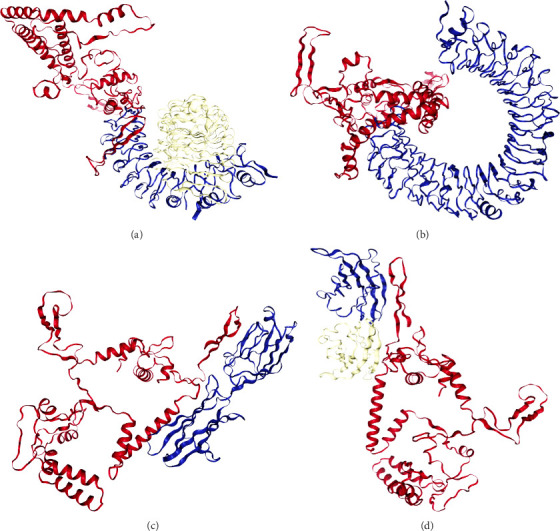
Docking complex of TLR2, TLR4, and B7 (1 and 2) with the vaccine construct. (a) TLR2, (b) TLR4, (c) B7-1, and (d) B7-2.

The results of the server (LZerD web server) showed that there are 10 possible models between the proposed vaccine and the TLR2, TLR4, B7-1, and B7-2 receptors. Based on the sum-rank score, the most stable model has been chosen (Table 6, Figure 5).

**Figure 6:**
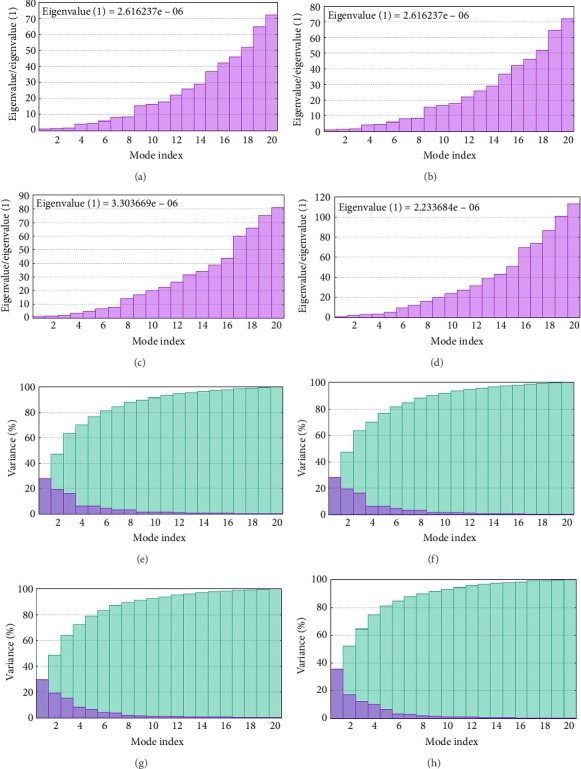
The eigenvalue plot represents the complex between the vaccine candidate and receptors using the iMODS server ((a) TLR2, (b) TLR4, (c) T7-1, and (d) T7-2). The variance plot (e–h) represents variance associated with the model. Green color refers to accumulative variance, while purple color represents individual variance of the models (e) TLR2, (f) TLR4, (g) B7-1, and (h) B7-2.

As shown in Figure 5, eigenvalues of the docked complexes were 2.61623437e^−6^, 2.2616237e^−6^, 5.303669e^−6^, and 2.233684e^−6^. These results indicate that four complexes have strong deformability. In Figure 6(b), the pure bar refers to the individual variation, while green bar represents accumulative variation. Analysis of the variance plot shows that the individual variance of successive mode exhibited a modest decline.

**Figure 7:**
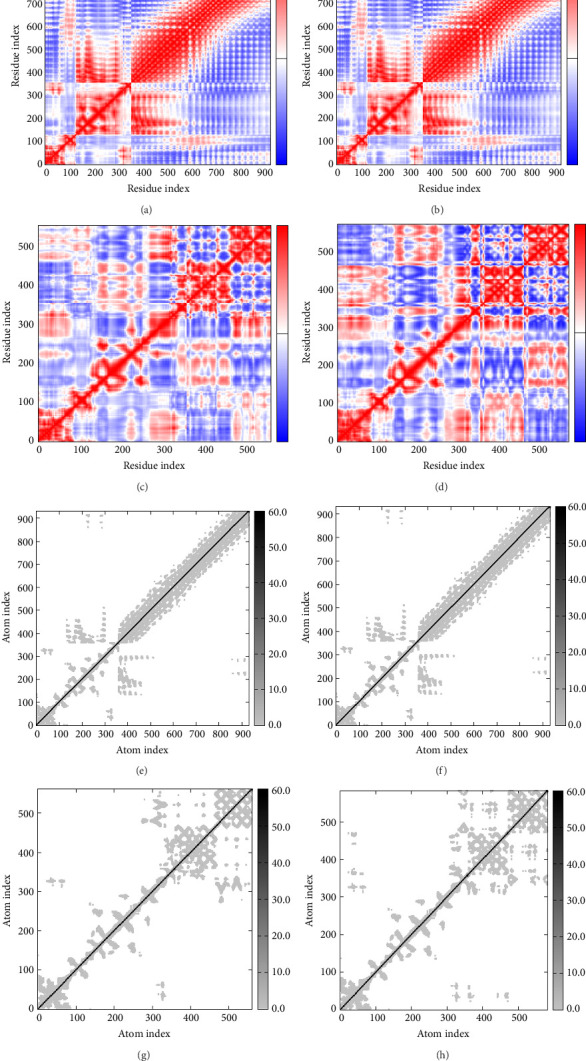
(a)–(d) Graph of the covariance matrix that represents complex formation by the vaccine candidate and receptors ((a) TLR-2, (b) TLR-4, (c) B7-1, and (d) B7-2) and the iMODS server. Red color represents correlated motion, while blue color and white color represent uncorrelated and anticorrelated motion, respectively. (e)–(h) The elastic network models that form by combination of the vaccine candidate with receptors ((a) TLR-2, (b) TLR-4, (c) B7-1, and (d) B7-2). Gray color represents the magnitude of the interaction.

In Figures 7(a), 7(b), 7(c), and 7(d), the degree of correlation within amino acid duplets scattered in dynamical regions was represented in different colors. The red color refers to correlated residue, and the blue color refers to noncorrelated residues, while white color representing anticorrelated amino acid duplets. In addition, the elastic network model that formed by combination of the vaccine with the receptors was generated to differentiate the atom pairs connected by springs (Figure 7(e), 7(g), 7(h)). Thus, the above results indicate that the vaccine-receptor complex has good stability and stiffness.

### 3.8. Immunostimulation

**Figure 8:**
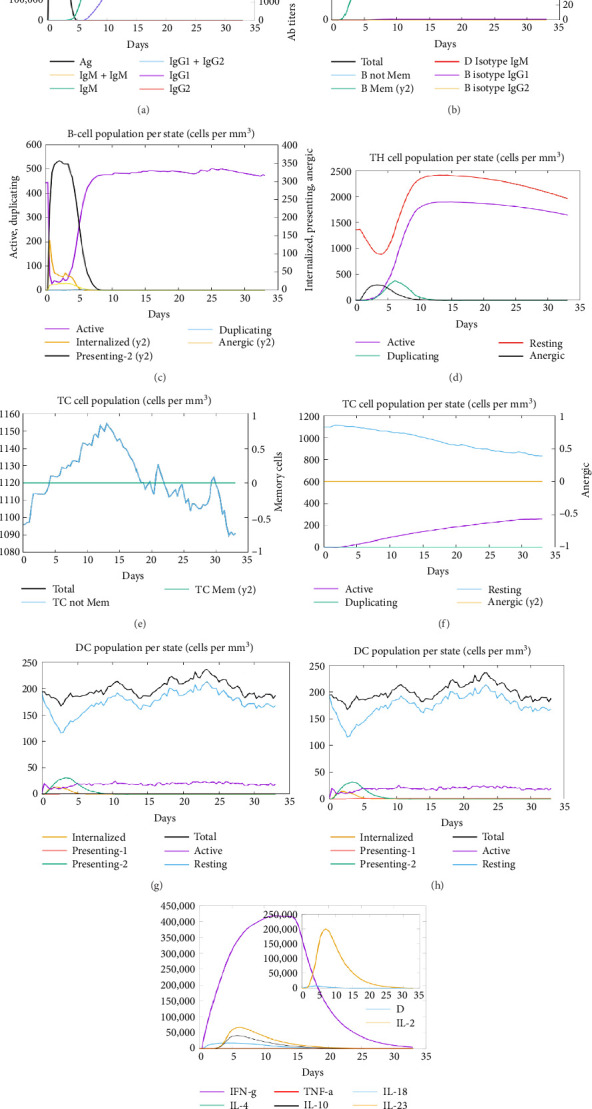
Immune simulation against proposed. (a) Antibody production postvaccine injection. (b) The population of the B-cell population after three injections. (c) The population of the B-cell per state. (d) The population of the helper tell. (e) The helper T-cell population per state. (f) The cytotoxic T-cell population per state. (g) The population of macrophage per state. (h) The population of the dendritic cell per state. (i) Production of cytokine and interleukin with the Simpson index of the immune response.

In the current study, predication of the immune response to three injections of the vaccine was performed using the C-ImmSim server (Figure 8(a)). In the 2end and 3ride injections of the vaccine, the concentrations of immunoglobulin M (IgM) and IgG continued to rise. B cells were observed and involved in the immune response, specifically in the humoral type. As shown in Figures 8(b) and 8(c), the population of B cells increases with three doses of the modeled vaccine. In the same manner, T cells (helper and cytotoxic) were also increased (Figures 8(d), 8(e), and 8(f)). In Figure 8(g), macrophages were enhanced after three doses of the vaccine, while dendritic cells remained stable (Figure 8(h)). Levels of IFN-γ and IL-2 cytokines were increased (Figure 8(i)). Finally, the low level of the Simpson index (D) refers to the diverse immune response. Overall, these findings indicated that the mRNA multiepitope vaccine induces a good immune response against *C. jejuni*.

### 3.9. Structural Versatility Assessment

**Figure 9:**
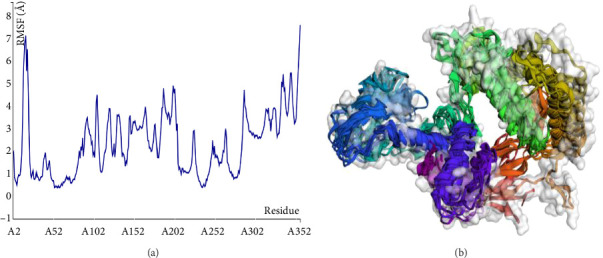
Root mean square fluctuation (RMSF) plot of the designed protein vaccine.

The structural versatility of the formulated vaccine was evaluated using CABS-flex, a modeling technique known for its rapid simulation abilities. The results presented in this section demonstrate that a substantial number of protein residues display favorable stability. The fluctuation profile of residues in the engineered protein vaccine has been illustrated in Figure 9 for reference.

### 3.10. mRNA Structure and Computerized Cloning

**Figure 10:**
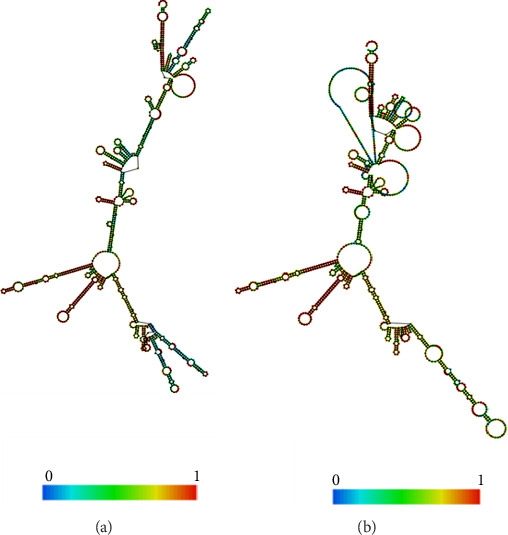
(a) Ideal secondary configuration. (b) Central secondary arrangement of the mRNA vaccine obtained through the RNAfold web server.

The RNAfold server was used to predict the structural arrangement of the mRNA (Figures 10(a), 10(b)). The results demonstrate that the mRNA vaccine is expected to exhibit stability, as evidenced by MFE of −419.90 kcal/mol for the structure. Additionally, the secondary centroid structure displayed a free energy of −329.50 kcal/mol (Figure 10(a)). The free energy of the thermodynamic ensemble was −440.61 kcal/mol (Figure 10(b)). These findings provide valuable insights into the thermodynamic robustness of the mRNA vaccine structure.

**Figure 11:**
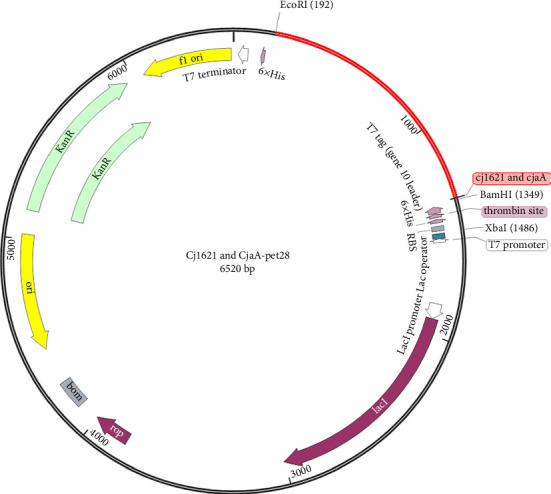
Design and in silico cloning of a modeled vaccine ligand into the pET21a(+) vector for enhanced expression.

Result of codon optimization shows that GC content of the proposed fragment was 68.2%, while CAI was 0.95. As shown in Figure 11, the proposed fragment was cloned in the vector (pET21) at position 198 and 1343 at the position of the selected restricted sites (*EcoRI* and *BamHI*).

## 4. Discussion

Using of in silico approaches to find multiple epitope vaccine is the effective way to control the increasing antibiotic resistance in pathogenic bacteria. The findings that presented in this study highlight significant advancements in the design of the multiepitope mRNA vaccine candidate against *C. jejuni*. The selected proteins, Cj1621 and CjaA, were evaluated for their antigenicity, epitope mapping, and subsequent construction of multiepitope vaccine. This demonstrates promising characteristics that warrant further exploration [37–40]. The VaxiJen v2.0 server's antigenicity assessment revealed strong recognition potential by the immune system, highlighting the viability of these candidates as potential vaccines. A thorough epitope analysis identified 15 immunogenic epitopes that are nontoxic and nonallergenic. These epitopes are capable to induce both B and T cell response, including 5 CTL epitopes, 6 HTL epitopes, 4 LBL epitopes, and one conformational B epitope. This diverse selection enhances the potential for a robust immune response.

The multiepitope vaccine was constructed with critical elements such as m7GCap, UTR (HBB), Kozak sequence, signal peptide, adjuvant (RpfE), and flexible linkers (EAAAK). This design promotes optimal folding and presentation of the epitopes. The physicochemical evaluation indicated a stable protein with a molecular weight of approximately 26,198.64 Da and a PI of 9.51, which suggests solubility under physiological conditions. Furthermore, estimated half-lives in various environments indicate vaccine's sustained effectiveness in mammalian cells, bacteria, and yeast. These findings collectively highlight the potential of our multiepitope vaccine candidate for advancing protective strategies against *C. jejuni* infections. Immunological analysis further confirmed that the candidate is nontoxic, nonallergic, and antigenic. These characteristics are essential for ensuring safety and efficacy in potential clinical applications. The stability and favorable properties of the chimeric protein underscore its suitability as a vaccine candidate. The 3D model of the vaccine construct was developed using trRosetta and refined with the Galaxy web server, resulting in a stable model with positive validation outcomes. Validation through SWISS-MODEL and ProSA confirmed the reliability and accuracy of the model for future studies. Moreover, molecular docking simulations with B7-1, TLR2, TLR4, and B7-2 receptors demonstrated the strong binding and stability of the complexes. The molecular dynamics analysis further supported the stability of these complexes, highlighting the deformability and favorable structural integrity of the interactions, essential for inducing an effective immune response. The immunostimulation analysis using the C-ImmSim server revealed a promising immune response elicited by the modeled vaccine and the increased amount of IgG and IgM antibodies. Also, proliferation of B and T cells indicates the ability of the vaccine to trigger both type of immune response (humoral and cellular). Additionally, the upregulation of cytokines and macrophages underscores the immunomodulatory properties of the vaccine construct. The CABS-flex analysis revealed that a substantial portion of protein residues in the engineered vaccine exhibits favorable stability, indicating promising structural characteristics crucial for vaccine efficacy. Moreover, the mRNA vaccine's structural prediction utilizing the RNA fold server indicated significant stability, evident by the minimum free energy values obtained. These complementary analyses offer a comprehensive understanding of the structural versatility and stability of both vaccine formulations, enhancing our insight into their potential effectiveness. Furthermore, the computerized cloning analysis demonstrated the suitability of the vaccine construct for good expression within the selected host, as evidenced by the favorable GC content and CAI-value. The presence of *Eco*RI and *Bam*HI sites for in silico cloning further facilitates the experimental validation and potential translation of the vaccine design into a practical setting. In this scientific domain, numerous studies have been carried out on a diverse range of infectious agents. The advancement of immunoinformatics and the advent of mRNA-based vaccines have led to the development of vaccines targeting pathogens such as *Yersinia*, *Brucella*, *Clostridium*, *Mycobacterium*, *Pseudomonas*, *Toxoplasma*, *Listeria*, and others [41–47]. The field of mRNA-based vaccine research has seen significant growth in response to these technological innovations, fostering new possibilities for combating infectious diseases.

## 5. Conclusion

In conclusion, the integrated computational approach utilized in this study not only provides valuable insights into the design and evaluation of a novel multiepitope mRNA vaccine but also lays the groundwork for potential experimental validation and clinical applications. This could be promising for a new avenue to combat *C. jejuni* infection [48].

## Figures and Tables

**Table 1:** Final selected CTL epitopes for the candidate vaccine.

Sr. no.	Epitopes	Antigenicity score	Toxicity	Allergenicity	Name of the gene	Conservancy (%)
1	FVAGVVFNV	0.6837	No	None	Cj1621	100
2	LQVNANTNI	1.0954	No	None	Cj1621	100
3	GVVFNVEFL	1.1249	No	None	Cj1621	100
4	QTPQRAEQV	0.7062	No	None	CjaA	100
5	YDETLKAHF	0.9790	No	None	CjaA	100

**Table 2:** Final HTL epitopes for the candidate.

Sr. no.	Epitopes	Antigenicity score	Toxicity	Allergenicity	Name of the protein	Conservancy (%)
1	EPNFSYKDGIQTPKG	0.7560	No	None	Cj1621	100
2	LEPNFSYKDGIQTPK	1.1344	No	None	Cj1621	100
3	LKNLQVNANTNIAPR	0.9771	No	None	Cj1621	100
4	NQGYDIALAKRIAKE	0.4231	No	None	CjaA	100
5	NNQGYDIALAKRIAK	0.4689	No	None	CjaA	100
6	SPYMKVALGVAVPKD	0.4887	No	None	CjaA	100

**Table 3:** LBL epitopes for the candidate.

No.	Start	End	Peptide	Length	Gene name	Toxicity
1	28	45	FLGKKDSNHDLYFSRNIE	18	Cj1621	Nontoxin
2	62	80	WANENLSSKITLDNSEKAE	19	Cj1621	Nontoxin
3	23	36	NSDSKTLNSLDKIK	14	CjaA	Nontoxin
4	49	54	KPPFGY	6	CjaA	Nontoxin

**Table 4:** Selected CBL epitopes for the candidate vaccine.

Residue	Location	Score
YKDGIQTPKGAAYLEPNFSYKDGIQTPKAAYLKN	76–109	0.752

**Table 5:** Immunogenic and physicochemical characteristics of the vaccine construct.

Properties	Measurement	Note
AA	357	Suitable
MW	26,198.64 Da	Appropriate
Theoretical pI	9.51	Basic
Formula	C_1719_H_2657_N_477_O_515_S_2_	—
Half-life (*Escherichia coli*)	10 h	—
Half-life (mammalian reticulocytes)	1.9 h	—
Half-life (yeast cells)	20 h	—
Grand average of hydropathicity	−0.587	Hydrophilic
Instability index of the proposed vaccine	25.91	Stable
Antigenicity	0.7381	—
Allergenicity	Nonallergic	—
Toxicity	Nontoxic	—

**Table 6:** Docking final model score.

Receptor	GOAP score	GOAP rank	DFIRE score	DFIRE rank	ITscore score	ITscore rank	Score RankSum
TLR2	−118570.90	40	−86935.85	90	−42791.22	11	141
TLR4	−114654.84	40	−80830.43	17	−40608.73	6	63
B7-1	−54628.14	124	−38140.12	34	−20752.52	5	163
B7-2	−55861.26	168	−40647.57	94	−22225.32	27	289

## Data Availability

The data that support the findings of this study are available on request from the corresponding author. The data are not publicly available due to privacy or ethical restrictions.
